# Mixed-Cropping Between Field Pea Varieties Alters Root Bacterial and Fungal Communities

**DOI:** 10.1038/s41598-019-53342-8

**Published:** 2019-11-18

**Authors:** Anthony Horner, Samuel S. Browett, Rachael E. Antwis

**Affiliations:** School of Science, Engineering and Environment, University of Salford, Salford, UK

**Keywords:** Agroecology, Ecological networks, Microbial ecology, Molecular ecology

## Abstract

Modern agricultural practices have vastly increased crop production but negatively affected soil health. As such, there is a call to develop sustainable, ecologically-viable approaches to food production. Mixed-cropping of plant varieties can increase yields, although impacts on plant-associated microbial communities are unclear, despite their critical role in plant health and broader ecosystem function. We investigated how mixed-cropping between two field pea (*Pisum sativum* L.) varieties (Winfreda and Ambassador) influenced root-associated microbial communities and yield. The two varieties supported significantly different fungal and bacterial communities when grown as mono-crops. Mixed-cropping caused changes in microbial communities but with differences between varieties. Root bacterial communities of Winfreda remained stable in response to mixed-cropping, whereas those of Ambassador became more similar to Winfreda. Conversely, root fungal communities of Ambassador remained stable under mixed-cropping, and those of Winfreda shifted towards the composition of Ambassador. Microbial co-occurrence networks of both varieties were stronger and larger under mixed-cropping, which may improve stability and resilience in agricultural soils. Both varieties produced slightly higher yields under mixed-cropping, although overall Ambassador plants produced higher yields than Winfreda plants. Our results suggest that variety diversification may increase yield and promote microbial interactions.

## Introduction

Agriculture is a key area for the security of human health and well-being, and productivity may need to increase two-fold by 2050 to provide sufficient food for our rapidly growing human population^1^. Modern farming practices such as intensive tillage and large-scale input of inorganic fertilisers have helped drive high agricultural outputs, but at the cost of widespread environmental degradation and ecosystem instability^2–5^. Furthermore, historic and continued domestication and selection of crop species have driven a severe reduction in genetic diversity in modern cultivars^6,7^. This intensive approach to agriculture has negatively affected biodiversity and associated ecosystem service provision at multiple biological levels, resulting in reduced stability and resilience in agricultural systems^8–13^. There is now a concerted call to focus efforts on developing sustainable, integrated management processes that minimise intensive practices and instead focus on ecological solutions to increasing food production^13–16^.

Plant-associated microbial communities are critical to host function and fitness^17,18^. Root and rhizosphere communities are particularly vital, and the composition of these has significant implications for nutrient dynamics, immune function, pathogen susceptibility, and stress tolerance, among other traits^19–23^. For example, mycorrhizal fungi and nitrogen fixing bacteria are responsible for around 80% of nitrogen and 75% of phosphorus assimilation by plants in temperate forests^24^. The vast array of benefits conferred by plant-associated microbial communities has made harnessing the plant microbiome a key area of agricultural research in recent years, as its potential to revolutionise sustainable agriculture is recognised^25–27^. Practices such as the addition of organic amendments to soil, the use of cover crops, increasing crop diversity in fields, and reducing soil disturbance have all shown to be effective at improving microbial diversity in soil, potentially reducing the need for resource inputs whilst maintaining high yields^13,14,16,28^. As such, a deeper understanding of the ecological functions of the plant-associated microbial communities may help in the development of new farming practices with reduced ecological damage.

There is considerable variation in rhizosphere composition between plant species, and concurrently, mixed-cropping between crop species can cause significant changes in microbial community composition and diversity, with implications for productivity^29–32^. Greater plant diversity also provides increased ecosystem service provision and associated biodiversity^33–36^ and thus, such as an approach may have benefits at multiple levels. Furthermore, different varieties or cultivars of the same crop also support different root-associated microbial communities^37–41^. A meta-analysis of 91 studies indicated that growing different varieties of the same crop together can increase yield and stability, particularly under biotic and abiotic stressors such as disease or weather variability^42^. Intra-cropping different varieties can also increase below- and above-ground invertebrate diversity^39^ [but also see^40^]. As such, mixed-cropping between different varieties of the same crop can improve crop performance and ecosystem service provision^16^. However, the effects of combining different crop varieties on microbial community composition are poorly understood.

Here we use a glasshouse pot experiment to identify how mixed-cropping between two field pea (*Pisum sativum* L.) varieties influenced yield and root bacterial and fungal communities. We hypothesised that mixed-cropping will alter bacterial and fungal communities in comparison to mono-cropped varieties, with concurrent increases in yield, microbial diversity, and species interactions.

## Methods

### Glasshouse experiment and yield data

The study was conducted in a greenhouse using two field pea varieties; Ambassador (Tamar Organics, UK) and Winfreda (Heritage Seed Library, UK), which were grown in 3 L pots containing potting soil (Moorland Gold, UK). We collected three samples of potting soil at the start of the experiment and froze these at −80 °C for subsequent analysis of microbial communities. We sowed two seeds per pot, equidistant from one another and the sides of the pots. Each pot contained either two Ambassador seeds, two Winfreda seeds, or one of each, with 8 pots per mono-crop group and 11 pots of the mixed-crop. Pots were randomly distributed across the growing space in the glasshouse, and plants were watered daily with tap water. The study ran for six weeks, during which time all plants flowered and produced pods. We then removed all pods and weighed these collectively to gain yield data for each plant. These data were not normally distributed and so to test for differences in yield between the four treatment groups (mono-cropped Ambassador, mixed-cropped Ambassador, mixed-cropped Winfreda, and mono-cropped Winfreda), we used a generalised linear model with quasipoisson distribution in the car package^43^, with a Tukey’s post hoc analysis conducted using the multcomp package^44^ in RStudio^45,46^.

### DNA extraction, 16S rRNA and ITS rRNA sequencing

At the end of the experiment, we isolated 250 mg of the root and associated soil close to the base of the above-ground part of the plant, and froze these samples at −80 °C until subsequent DNA extraction. We extracted DNA from root and soil samples using the Qiagen PowerSoil Pro kit (Qiagen, UK) according to the manufacturer’s instructions and quantified it using the high sensitivity assay kit on a Qubit^TM^ 3.0 Fluorometer (ThermoFisher Scientific, UK). We conducted 16S rRNA gene amplicon sequencing of the V4 region using dual indexed forward and reverse primers according to Griffiths & Harrison *et al*.^47^ and Kozich *et al*.^48^, and ITS rRNA gene sequencing according to Nguyen *et al*.^49^. For 16S rRNA amplification, we ran PCRs in duplicate using Solis BioDyne 5x HOT FIREPol^®^ Blend Master Mix, 2 μM primers and 1 μl of sample DNA, with thermocycling conditions of: 95 °C for 15 minutes; 28 cycles of (95 °C for 20 s, 50 °C for 60 s, 72 °C for 60 s) and a final extension at 72 °C for 10 minutes. For ITS rRNA gene amplification, we used 4ul of DNA with thermocycling conditions of 95° for 10 minutes; 35 cycles of (95° for 30 s, 54° for 45 s, 72° for 60 sec) and a final extension at 72° for 10 minutes. We combined PCR replicates and cleaned these using HighPrep^TM^ PCR clean up beads (MagBio, USA) according to the manufacturers’ instructions. We quality checked products using the Agilent 2200 TapeStation and quantified these on a Qubit^TM^ 3.0 Fluorometer. Samples were then pooled to equimolar concentrations. ITS and 16S rRNA gene amplicon sequencing was conducted on two separate runs using paired-end reads (2 × 250 bp) with v2 chemistry on the Illumina MiSeq platform at the University of Salford. Negative (extraction blanks) and positive (mock community) controls were included in the sequencing run.

### Pre-processing of amplicon sequencing data

We conducted all analyses in RStudio (v1.0.153)^46^ for R (v3.4.1)^45^. We processed 16S rRNA and ITS rRNA gene amplicon sequences in DADA2 v1.5.0^50^ using the default pipelines. A total of 4,057,971 raw sequence reads from 64 samples were generated from 16S rRNA gene sequencing, and 881,602 from ITS rRNA gene sequencing. Once paired end reads were merged, modal contig length was 253 bp (range of 240–260 bp) for 16S rRNA data and 219 bp (range of 210–434) for ITS rRNA data. For bacteria, we removed sequence variants (SVs) with length > 260 bp (46 out of 8516 SVs; 0.005% of total sequences) along with chimeras and two SVs found in the negative controls. As the ITS rRNA gene region is highly variable (Lindahl *et al*. 2013), we did not filter sequence variants based on length but we did remove chimeras and one SV identified in the negative controls. DADA2 identified all expected unique SVs in both the bacterial and fungal mock communities.

SVs with fewer than 100 reads across all samples were removed, leaving a median of 40870 SVs per sample (range of 14020–95216) for 16S rRNA data and 5684 SVs per sample (range of 2653–13954) for ITS rRNA data. We assigned 16S taxonomy using the SILVA v132 database^51,52^ and ITS taxonomy using the UNITE v7.2 database^53^ and exported the final SV tables, taxonomy tables and sample metadata to the phyloseq package^54^ for further analysis. To provide greater taxonomic detail about unidentified SVs and to stop the removal of these during analyses that agglomerate to a given taxonomic level, we fully annotated the taxonomy tables to species level using higher levels assignments (e.g. SV3 was named “Family_Prevotellaceae” at the genus and species levels).

### Root community analyses

We constructed stacked plots to visualise the relative composition of root and soil bacterial and fungal communities at the class level according to treatment group (mono-cropped Ambassador, mixed-cropped Ambassador, mixed-cropped Winfreda, and mono-cropped Winfreda). We visualised the variation in the relative abundance of root communities of bacterial and fungal communities using NMDS plots (with Bray-Curtis distance) in phyloseq. We used a permutational ANOVA (PERMANOVA; adonis) in the vegan package^55^ to identify variation in the bacterial and fungal root communities according to treatment group. We determined the effect of variety, cropping type and their interaction on the relative abundance of the top ten bacterial and fungal families using one-way ANOVAs, and visualised this variation using box plots. We further visualised variation between treatment groups by plotting the relative abundance of genera as a heatmap (only bacterial genera with a combined relative abundance of > 1% across all four treatment groups were plotted, whereas all fungal genera were included). We calculated alpha-diversity (species richness) across the two microbial kingdoms by subsampling the raw SV count table to a standardised number of reads (equal to the sample with the lowest number of reads) using an iterative approach (100 times), averaging the diversity estimates from each trial. We tested for differences according to treatment group using a general linear model with quasipoisson distribution.

To identify relationships between root fungal and bacterial communities, we extracted Jensen-Shannon divergence and Jaccard distance matrices between all samples for both fungal and bacterial communities in the phyloseq.^54^ and vegan^55^ packages. We used mantel tests to correlate fungal and bacterial community distances for each measure separately. We rarefied the bacterial and fungal data sets to the same size as the sample with the lowest number of reads (13332 reads for bacteria and 1824 reads for fungi). We agglomerated these to genus level and then merged the phyloseq objects for bacterial and fungal communities for each plant. Using these cross-kingdom data, we calculated the co-occurrence between each pair of microbial genera by constructing a Spearman’s correlation coefficient matrix in the bioDist package^56,57^. We calculated the number of interactions with p < 0.05 for each treatment, in addition to those with −0.50 > rho > 0.50, and −0.75 > rho > 0.75 as indicators of network stability. We visualised those with rho > 0.75 (strong positive interactions) and rho < −0.75 (strong negative interactions) for the four treatment groups separately using network plots produced in igraph^58^.

## Results

### Yield data

**Figure 1:**
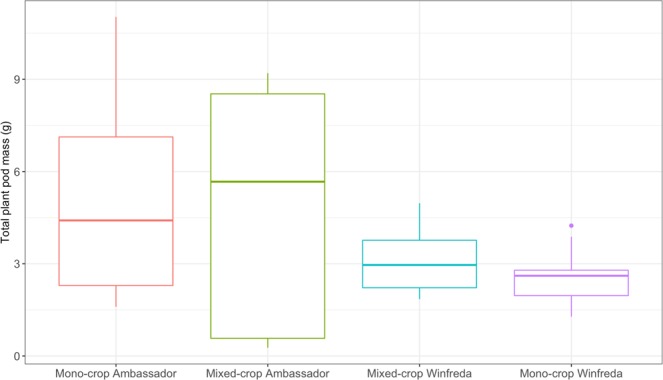
Median average (with 25% and 75% quartiles) of the total pod yields for Ambassador and Winfreda pea plants grown using a mono-cropping or mixed-cropping strategy.

There were significant differences in pod yield between the four treatment groups (X^2^ = 17.436, d.f. = 3, p < 0.001). Tukey’s post hoc analysis showed the yield for the mono-cropped Winfreda plants was significantly lower than that of mono-cropped Ambassador plants (p = 0.002) and mixed-cropped Ambassador plants (p = 0.009; Fig. 1). In general, yields were higher for the Ambassador variety than Winfreda, regardless of whether these were grown as mixed- or mono-crop, although yields were much more variable for Ambassador (Fig. 1). The average yield of each variety was higher when grown as a mixed-cropped than a mono-crop (Fig. 1), although these were not statistically significant (all p > 0.05).

### Bacterial community composition

The taxonomic composition of bacterial communities in the initial experimental soil was different to those of pea plant roots, although it had similar classes represented but at different proportions (Fig. S1). Across both soil and roots, the main bacterial classes present were Actinobacteria, Alphaproteobacteria, Anaerolineae, Bacilli, Bacteroidia, Clostridia, Deltaproteobacteria, Gammaproteobacteria, and in plant roots only, Verrucomicrobiae (Fig. S1).

**Figure 2:**
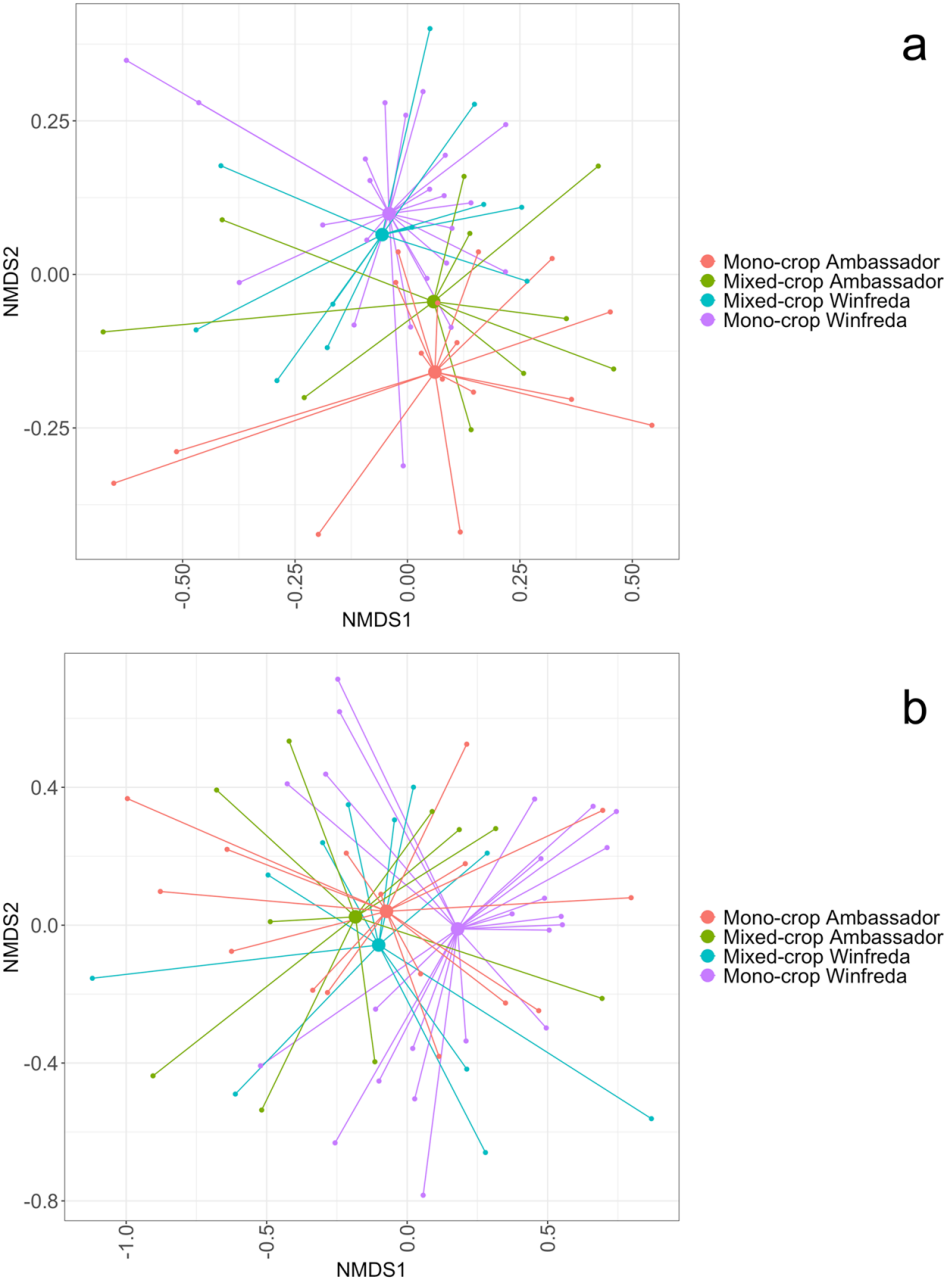
NMDS plots of (**a**) bacterial and (**b**) fungal communities associated with the roots of Ambassador and Winfreda pea plants grown using a mono-cropping or mixed-cropping strategy. Smaller dots indicate individual samples and larger dots represent the group average.

The taxonomic composition of the roots of pea plants were significantly different according to treatment group (F_3,56_ = 2.423, R^2^ = 0.078, p = 0.001; Fig. 2a). The root bacterial communities of the two varieties grown as mono-crops were most different to one another (Fig. 2a). The roots of the Winfreda variety had similar bacterial community compositions regardless of whether they were mono-cropped or mixed-cropped (Fig. 2a). The roots of the mixed-cropped Ambassador variety had a root bacterial community composition that was intermediate between that of mono-cropped Ambassador plants and Winfreda plants (Fig. 2a).

**Figure 3:**
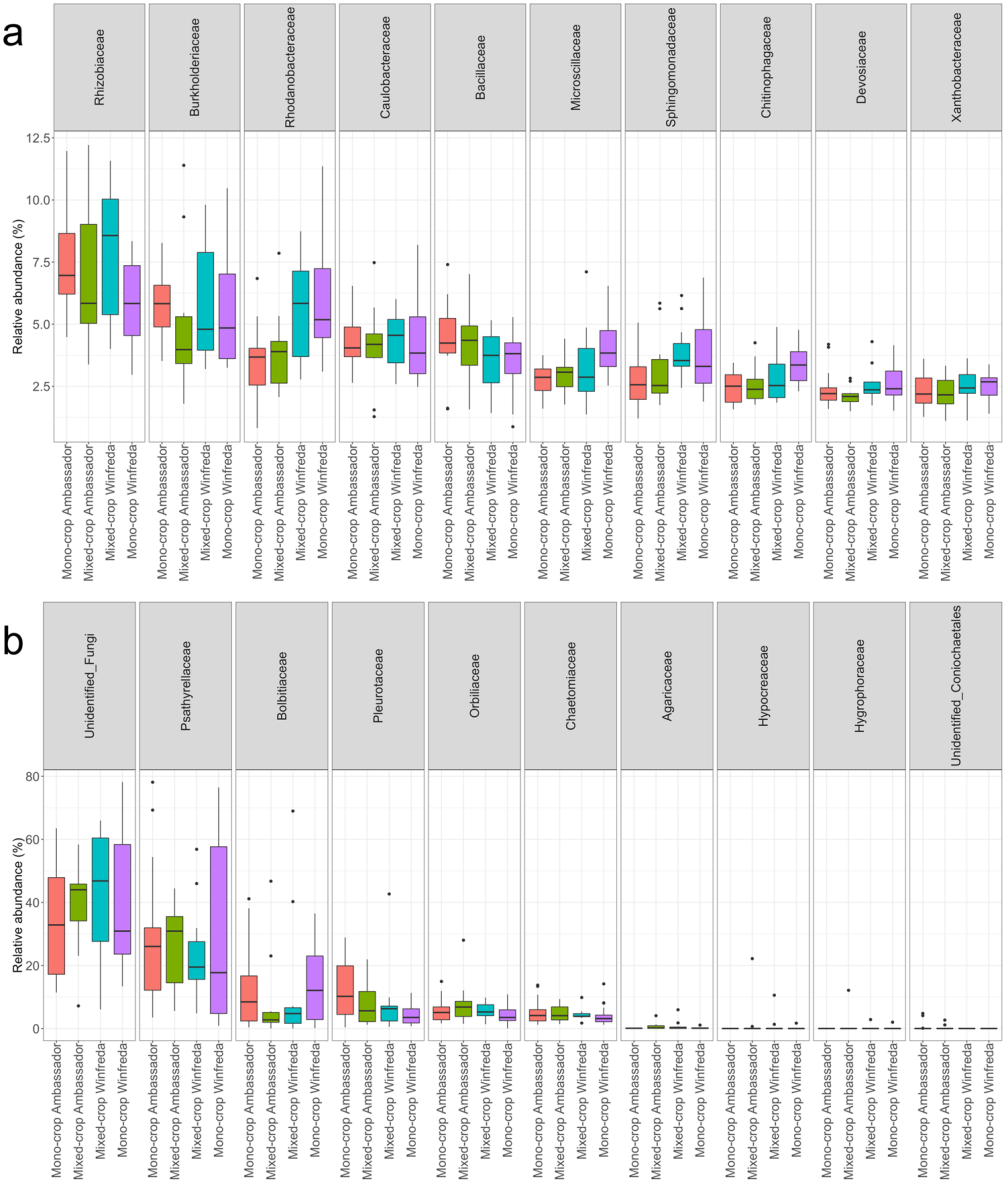
Relative abundance of the 10 most abundant bacteria (**a**) and fungi (**b**) in the roots of two pea varieties under two different cropping strategies.

The ten most abundant bacterial families in the roots of the two pea varieties are shown in Fig. 3a. Variety had a significant effect on the relative abundance of five of the ten most abundant root bacterial families (Rhodanobacteraceae, Bacillaceae, Microscillaceae, Sphingomonadaceae, Chitinophagaceae), and the interaction between variety and cropping strategy had a significant effect on one (Rhizobiaceae) (all p < 0.05). In the case of Rhizobiaceae, mixed-cropping led to an increase in abundance of this family in the roots of Winfreda plants compared with its mono-cropped counterpart. Similar variation between treatment groups in the relative abundance of taxa is evident at the genus level (Fig. S2).

### Fungal community composition

As with bacterial communities, the fungal community of soil at the start of the experiment was considerably different to that of pea plant root communities, with a relatively even distribution of fungal classes (Fig. S3). Four of the most dominant fungal classes in soil were also the most dominant in root communities; Agaricomycetes, Orbiliomycetes, Sordariomycetes and a group of unidentified fungi (Fig. S3). Treatment group had a significant effect on root fungal community composition (F_3,56_ = 1.618, R^2^ = 0.078, p = 0.024; Fig. 2b). The average root fungal communities of mono-cropped Winfreda pea plants were different to those of the other three treatment groups, which clustered together closely (Fig. 2b). Thus, converse to bacterial communities, root fungal communities of the Ambassador variety remained stable despite mixed-cropping, whereas those of the Winfreda variety plants became more similar to Ambassador under mixed-cropping (Fig. 2b).

The ten most abundant fungal families in the roots of the two pea varieties are shown in Fig. 3b. There was a significant effect of variety on the relative abundance of two of the ten most abundant root fungal families (Pleurotaceae and unidentified Coniochaetales), and cropping strategy had a significant effect on one (Agaricaceae) (all p < 0.05). Some amount of variation in the relative abundance of taxa between treatment groups is also evident at the genus level (Fig. S4). In contrast to the large number of bacterial genera identified through amplicon sequencing, relatively few fungal genera were identified (Fig. S4).

### Cross-kingdom interactions

There were no significant differences in total microbial SV richness between treatment groups (F_3,56_ = 0.841, p = 0.477; Fig. 5). There was a significant relationship between fungal and bacterial communities of roots for both Jensen-Shannon divergence (JSD; r = 0.198, p = 0.003) and Jaccard’s distance measures (r = 0.144, p = 0.001). As fungal community distance increased, so did bacterial community distance, or in other words, roots with more similar fungal communities also had more similar bacterial communities (Fig. S6).

**Figure 4:**
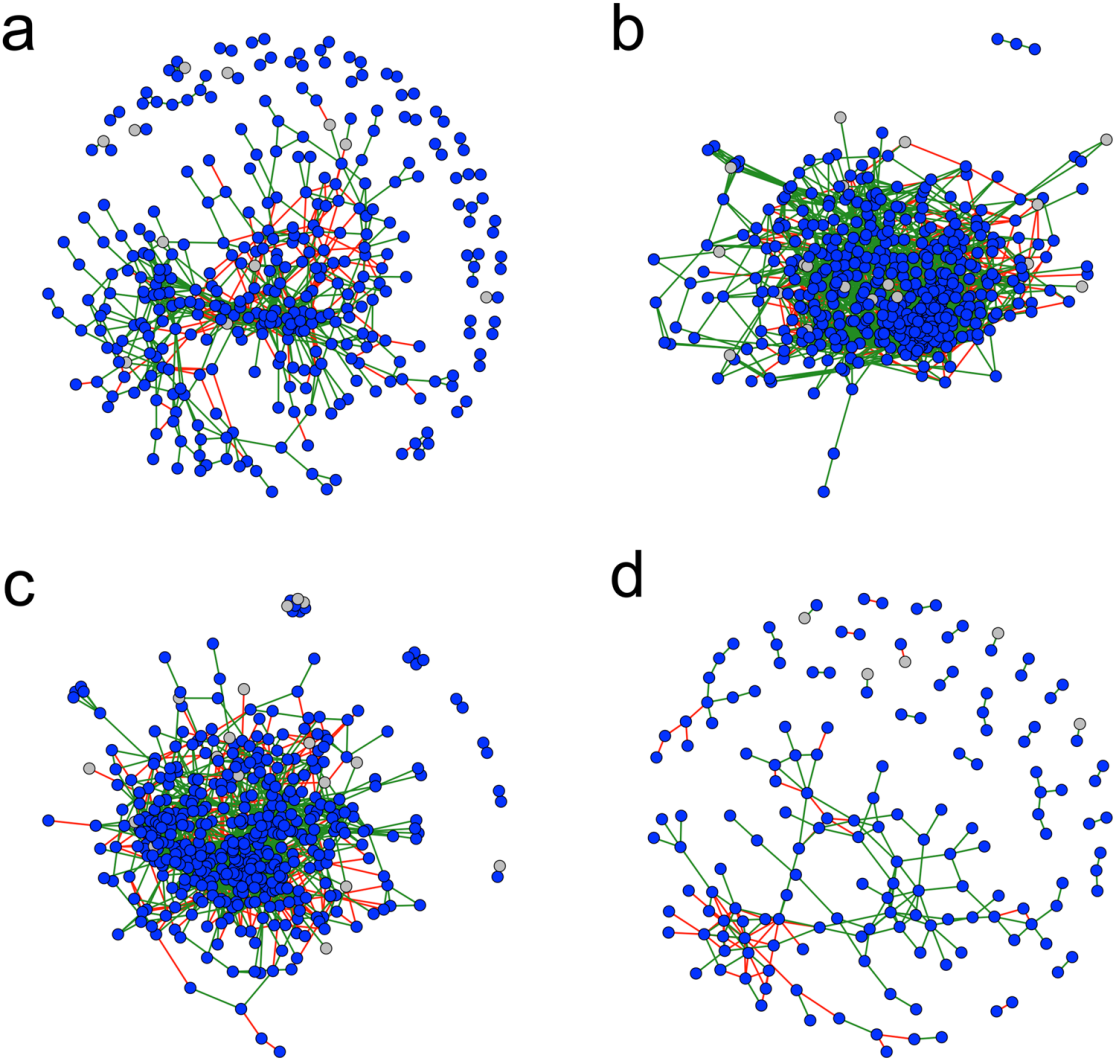
Co-occurrence networks showing significant (p < 0.05) positive (rho > 0.75; green edges) and negative (rho < −0.75; red edges) interactions between bacterial (blue nodes) and fungal (grey nodes) in the roots of (**a**) Winfreda pea plants grown as a mono-crop; (**b**) Winfreda pea plants mixed-cropped with Ambassador pea plants; (**c**) Ambassador pea plants mixed-cropped with Winfreda pea plants; and (**d**) Ambassador pea plants grown as a mono-crop.

Co-occurrence analysis revealed that the roots of both pea varieties had a considerably greater number of statistically significant (p < 0.05) microbial interactions when mono-cropped than mixed-cropped (Table 1). Both varieties had larger networks (i.e. more microbial interactions) under a mono-cropping strategy than a mixed-cropping strategy, however the majority of these were relatively weak (i.e. −0.75 < rho < 0.75). When considering only strong (i.e. −0.75 > rho > 0.75) or strong positive (rho > 0.75) interactions, both varieties formed larger, stronger networks under a mixed-cropping strategy (Table 1). Approximately 70% of these interactions were positive (Table 1; Fig. 4a–d). The majority of microbial interactions were between bacteria, although fungal genera were also involved in both positive and negative interactions (Table 1; Fig. 4a–d).

**Table 1:** Number of statistically significant (p < 0.05) microbial interactions (proportions in brackets) between microbial genera in the roots of two field pea varieties under two cropping strategies. Results shown for various correlation strengths (rho).

Treatment	Number of interactions	Number of interactions (−0.50 > rho > 0.50)	Number of interactions (−0.75 > rho > 0.75)	Number of interactions (rho > 0.75)
Mono-crop Ambassador	11952	11303 (95%)	762 (6%)	531 (4%)
Mixed-crop Ambassador	9969	9969 (100%)	3553 (36%)	2538 (25%)
Mixed-crop Winfreda	9419	9419 (100%)	2229 (24%)	1556 (17%)
Mono-crop Winfreda	13615	6107 (49%)	195 (1%)	141 (1%)

## Discussion

Here we demonstrate that mixed-cropping between two varieties of field peas alters root microbial community composition in comparison to cropping with single varieties (mono-cropping). The two varieties supported significantly different root bacterial and fungal communities to one another when mono-cropped. Under mixed-cropping, both fungal and bacterial communities tended to shift towards a composition that was intermediate to those of mono-cropped plants. Both bacterial and fungal growth and reproduction are dependent on nutrient type, quality and quantity^59,60^, and nutrient uptake and exudate deposition often varies between crop varieties^61–64^. Thus, changes in microbial communities may reflect differences in plant exudates produced under mixed-cropping in comparison to mono-cropping. There is growing evidence that plants, including crops, can recognise the relatedness of neighbours, possibly through root exudates or rhizosphere communities, and respond with changes in phenotype, resource use, exudate production, and gene expression^65–69^. Here, we provide novel evidence that plant root microbial communities also respond to the relatedness of their neighbours, although the mechanisms for this are not clear.

There were differences between the two varieties in the responses of bacterial and fungal communities to mixed-cropping. Under mixed-cropping, root bacterial communities of Ambassador shifted whilst those of Winfreda remained stable. Conversely, root fungal communities of Winfreda shifted under mixed-cropping, whilst those of Ambassador remained stable. That root fungal and bacterial communities of the two varieties responded differently to mixed-cropping suggests they are using plant- or microbe-derived carbon differently, or that the two kingdoms are undergoing niche partitioning. For example, there is evidence of variation in resource use for different taxonomic groups of bacteria and fungi, particularly in relation to nitrogen and carbon^70–73^. Although the two kingdoms do not necessarily exploit entirely different energy channels^60^, broadly speaking, bacteria are primarily considered r-strategists; fast-growing organisms that thrive in nutrient-rich, unstable environments with a reliance on labile carbon^59,60,74^. On the other hand, fungi tend to adopt both an r-strategy as well as a more a slow-growing K-strategy, making use of stable, low-nutrient environments and consuming both labile and recalcitrant carbon^59,60,74^. Thus, differences in microbial community structure are likely to reflect variation in exudate-derived carbon and/or nitrogen sources arising from the interaction between crop variety and cropping strategy. Despite varietal differences in the responses of bacterial and fungal communities to mixed-cropping, we found a broad, significant, correlation between the composition of the two microbial kingdoms in pea roots. Although these cross-kingdom interactions remain relatively under-studied, their role is of growing interest^75^. For example, cross-kingdom interactions may be important for biofilm production^76,77^, and fungal communities can influence bacterial colonisation through modulation of carbon, nitrogen, and pH^78,79^. Indeed, community composition may also be determined, in-part, through microbially-derived carbon production and other forms of interaction^59,66,80,81^. Soil pH also has a considerable effect on bacterial community composition, and a lesser effect on fungal communities^82,83^. Therefore, a greater understanding of how cropping strategies influence exudate production, nutrient cycling, and soil parameters may provide insight into the mechanisms driving community changes.

The co-occurrence results show that root communities form weaker networks under mono-cropping in comparison to mixed-cropping. This suggests plants provide a less stable environment for microbial community development when planted as a genetic monoculture. One might expect more unstable environments to arise from competition between crop varieties^84^, however, our results suggest mixed-cropping may promote larger, more stable microbial networks. This may have implications for a range of host traits and environmental aspects, such as yield output, pathogen resistance, carbon deposition, and soil fertility, to name a few^16,60^. However, in our study, mixed-cropping did not lead to increases in microbial diversity, which would likely be of benefit to agricultural soils and associated biodiversity^5,16^. Our results also show that in some cases, mixed-cropping introduced microbes to the roots of the other variety, and in other cases, some microbes appear in mixed-cropped plants without necessarily being introduced by the other variety. This further supports the hypothesis that mixed-cropping leads to novel exudates that alter microbial community composition. Further work is required to understand the function of the different members of the root microbial community, and how different varieties can be combined to facilitate the development of communities that maximise improvements to food production and carbon storage, among other desirable attributes for agricultural systems^16^.

Root microbial communities at the end of the study were considerably different to the microbial communities associated with the soil in which they were grown, albeit with similar groups, indicating that pea plants select their microbial communities from their environment. This is a well-known phenomenon in plants known as the ‘rhizosphere effect’ (reviewed in^74^). Previous studies have also shown that the microbial communities that inhabit the rhizosphere are significantly different in the early development stages compared with the latter vegetative, flowering, bolting, and senescence stages, during which times, microbial communities tend to converge^85–88^. This is likely linked to changes in root secretions, which increase throughout development with higher root secretion of sugars in early growth stages, which are then replaced by phenolics and specific amino acids in late growth stages, with a reduction in secretions post-flowering^64,88–90^. We collected root samples after flowering so that we could also obtain yield data, which suggests we sampled the end-state microbial communities rather the earlier, dynamic communities, which may have shown more pronounced differences according to variety and cropping strategy.

We found much larger bacterial than fungal communities associated with the roots of field peas. As a legume and a member of the Fabaceae family, peas have a strong association with rhizobia bacteria, which play an important role in nitrogen fixation, particularly in nutrient-poor soil^19^. Furthermore, bacteria are expected to dominate over fungi in high-nutrient environments^59,91,92^. Thus, the use of high-quality compost rather than nutrient-poor soil in this study is likely to have affected the community composition and may further explain the relatively low fungal diversity identified in our study. In addition, soil composition and structure also influence root architecture, exudate production and plant signalling, with implications for microbial communities^19,93–95^. Therefore, the response of microbial communities of peas (and other crops) to mixed-cropping may be even more pronounced in nutrient-depleted or highly-disturbed soils, as are commonly associated with agricultural fields. Indeed, in such soils, the use of different crop genotypes may be a critical tool for maximising the availability of nutrients for crop growth^95^. The effects of mixed-cropping on nutrient quality of the crop would also be of considerable interest for human health^96,97^.

Although Ambassador plants had significantly higher yields than Winfreda, we found that mixed-cropping increased the average yield of both varieties relative to its mono-cropped counterpart. Plants may be benefitting from mixed-cropping via release from negative plant-soil feedbacks conferred by mono-cropped rhizospheres^66,98,99^, or through altered carbon translocation^100^. Thus, mixed-cropping between varieties may have the potential to increase yields through selection of complimentary varieties that promote positive plant-soil feedback relationships or carbon assimilation. A meta-analysis of 91 intercropping studies found a negative correlation between nutrient content of soil and the net yield benefit from the use of intra-cropping^42^, compared with soils high in nutrients, which resulted in low to no yield increases. This suggests that when nutrient content of the soil is low, a higher yield benefit from intercropping is likely. This may be attributed to a greater level of organic matter mineralisation by soil microbes when plants are mixed-cropped, increasing nutrient availably to plants^42,101^. Thus, greater improvements to yields arising from mixed-cropping strategies may also be more evident in nutrient-poor soils. Further trials with more genotypes, particularly under field settings, may identify complimentary varieties that maximise yields and restore microbial diversity to agricultural landscapes^16^. Given that there is a heritable basis to root exudates^93^ and microbial communities^38,102^, such studies would ideally be conducted in combination with host genotype analyses across the spectrum of host diversity. Furthermore, the implications of such cropping strategies for associated biodiversity and ecosystem service provision are a particularly important aspect to consider.

In conclusion, we show that mixed-cropping between two field pea varieties leads to differential changes in bacterial and fungal community composition of roots. This may be related to changes in soil chemistry or microbe-microbe interactions, or result from differences in plant exudate production arising from inter-variety competition. Mixed-cropped plants supported larger, more stable microbial co-occurrence networks, suggesting this approach may improve resilience and stability of root-associated communities and subsequently, the crop. More work is required to identify mechanistic drivers of variation in microbial communities arising from mixed-cropping, in addition to combinations of genotypes that can maximise yield production, disease resistance, and wider ecosystem provision in agricultural settings. Such an approach may also provide an opportunity to restore microbial diversity to degraded agricultural ecosystems^103,104^.

## Supplementary information

Supplementary material

Supporting Information

## Data Availability

All sequencing data produced during this study are available on the NCBI SRA database under project numbers PRJNA505960 and PRJNA526546. All analysis code is available on request from Rachael Antwis.
