# Supplementary material for: Mixed-Cropping Between Field Pea Varieties Alters Root Bacterial and Fungal Communities
Source: Sci Rep. 2019 Nov 18;9:16953. doi: 10.1038/s41598-019-53342-8 (PMC6861290; doi:10.1038/s41598-019-53342-8)
Supplement: Supplementary file 1 — Supplementary material [file 41598_2019_53342_MOESM1_ESM.docx]

# **SUPPLEMENTARY MATERIAL:**

# **MIXED-CROPPING BETWEEN FIELD PEA VARIETIES ALTERS ROOT BACTERIAL AND FUNGAL COMMUNITIES**

Anthony Horner, Samuel S. Browett and Rachael E. Antwis

University of Salford, UK, [Rachael.Antwis@gmail.com](mailto:Rachael.Antwis@gmail.com)


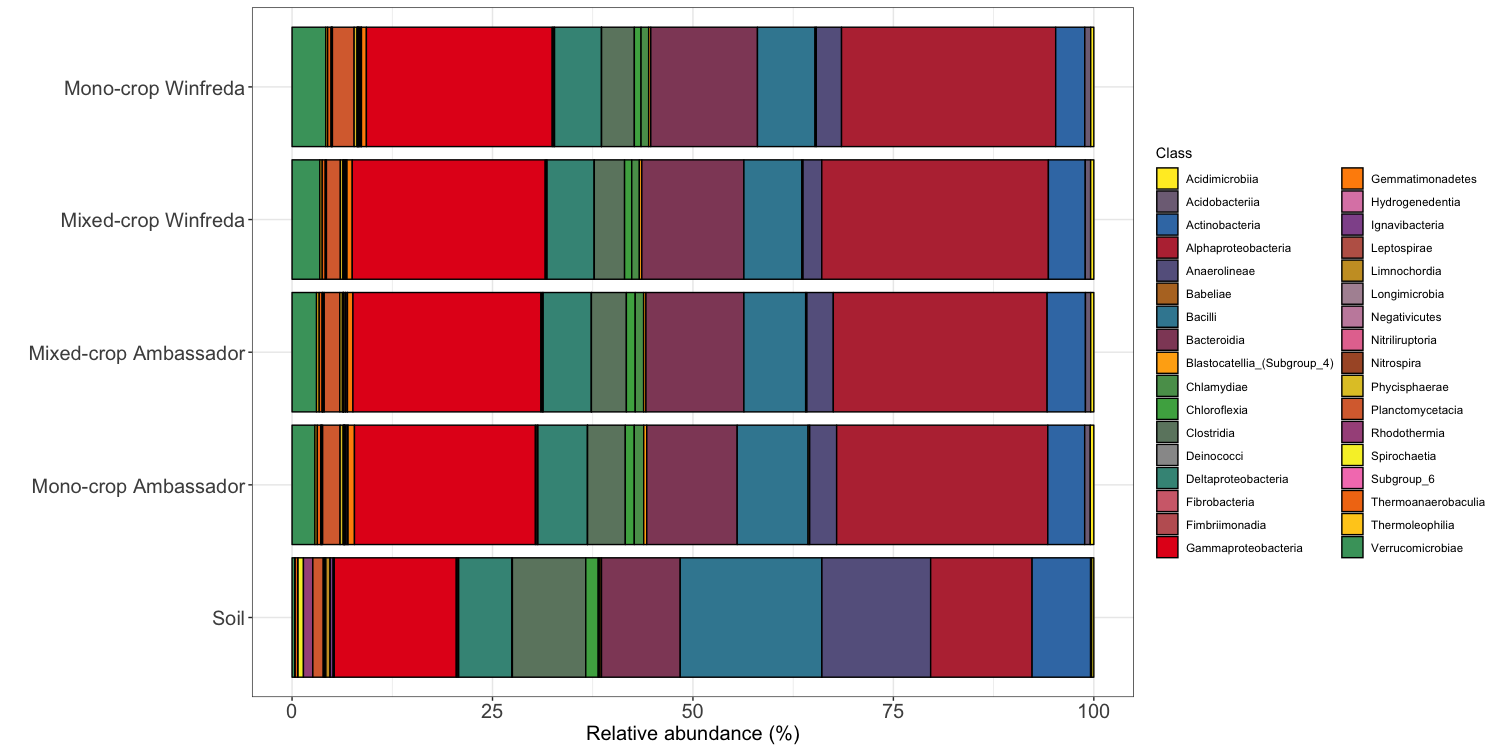


**Fig. S1**

Relative abundance of bacterial classes associated with the soil at the start of the experiment, and with the roots of two varieties of pea plants grown under a mono-cropping or mixed-cropping strategy at the end of the study.


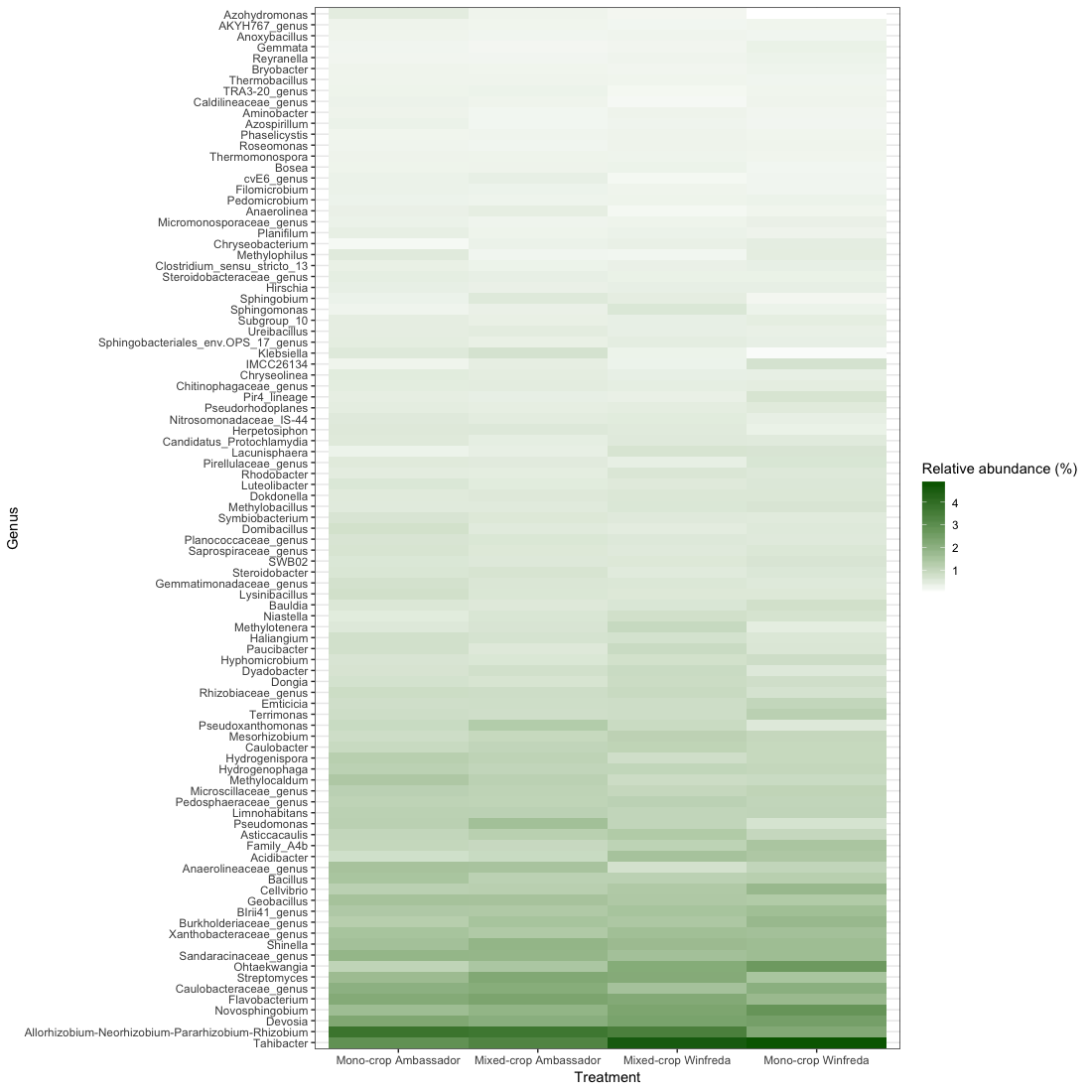


**Fig. S2**

Relative abundance of bacterial genera associated with the roots of two varieties of pea plants grown under a mono-cropping or mixed-cropping strategy (only those than a combined relative abundance of >1% across all treatment groups are shown).


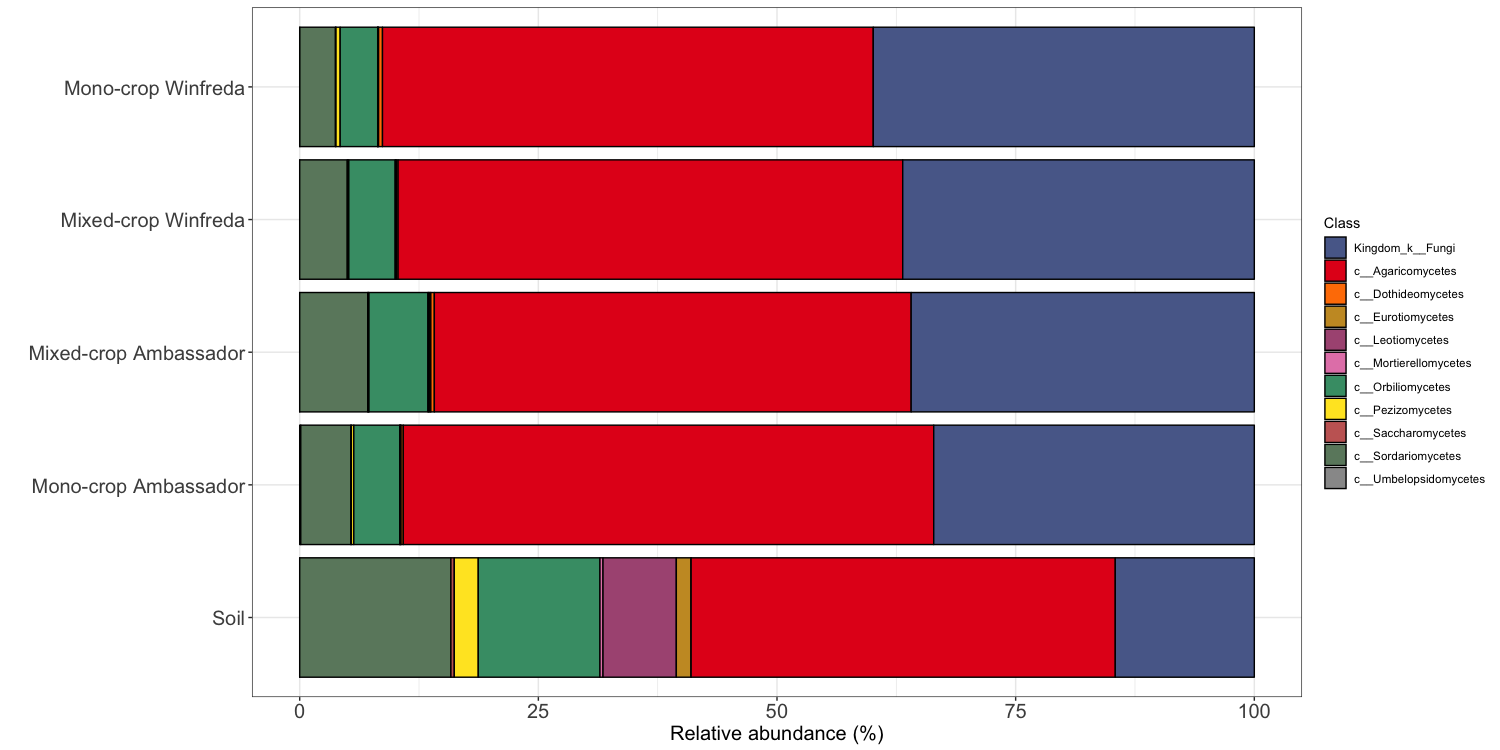


**Fig. S3**

Relative abundance of fungal classes associated with the soil at the start of the experiment, and with the roots of two varieties of pea plants grown under a mono-cropping or mixed-cropping strategy at the end of the study.


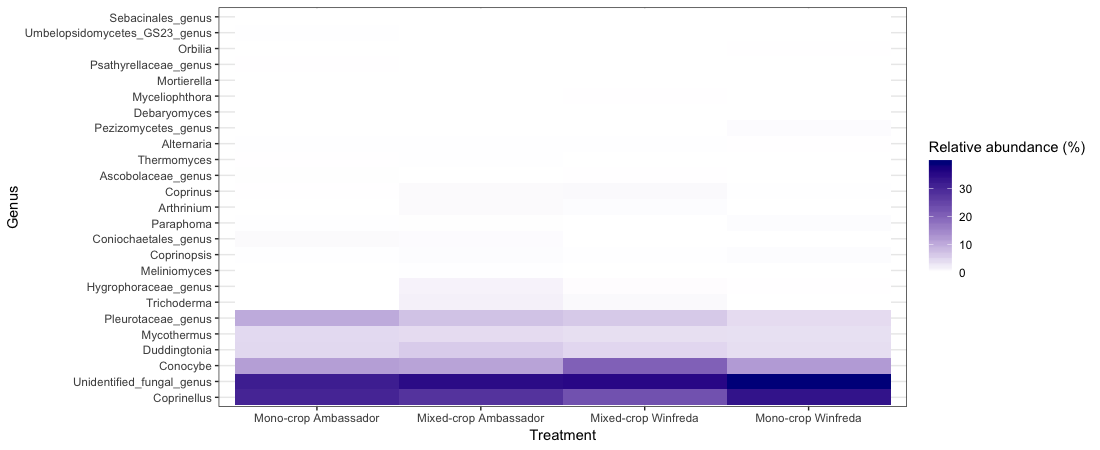


**Fig. S4**

Relative abundance of fungal genera associated with the roots of two varieties of pea plants grown under a mono-cropping or mixed-cropping strategy.


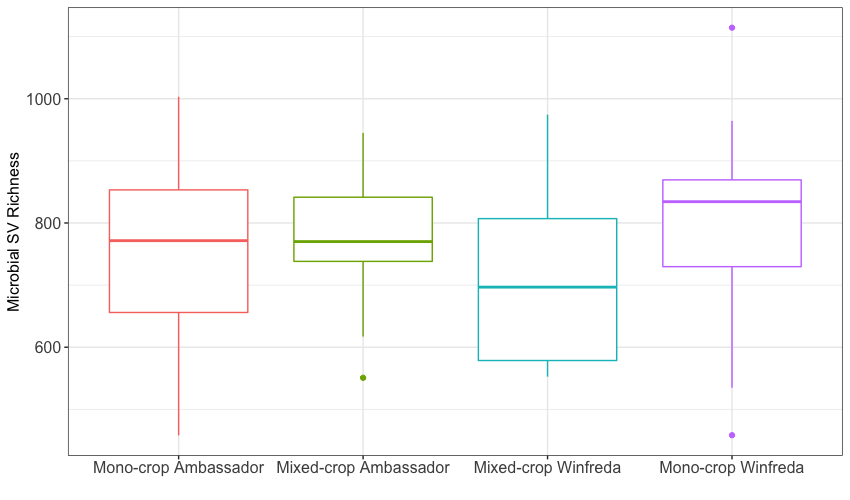


**Figure S5**

Microbial sequence variant richness (median average with 25% and 75% quartiles) associated with the roots of pea plants in the four different treatment groups.


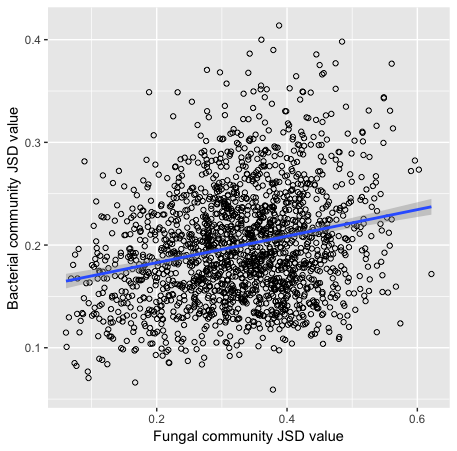


**Figure S6**

Relationship between Jensen-Shannon Divergence (JSD) values for fungal and bacterial communities associated with the roots of two varieties of pea plants.
